# Genome-wide linkage analysis for alcohol dependence: a comparison between single-nucleotide polymorphism and microsatellite marker assays

**DOI:** 10.1186/1471-2156-6-S1-S8

**Published:** 2005-12-30

**Authors:** Qianli Ma, Yi Yu, Yan Meng, John Farrell, Lindsay A Farrer, Marsha A Wilcox

**Affiliations:** 1 Genetics Program, Boston University School of Medicine, 715 Albany Street, Boston, MA 02118, USA; 2 Bioinformatics Graduate Program, Boston University, 44 Cummington Street, Boston, MA 02215, USA

## Abstract

Both theoretical and applied studies have proven that the utility of single nucleotide polymorphism (SNP) markers in linkage analysis is more powerful and cost-effective than current microsatellite marker assays. Here we performed a whole-genome scan on 115 White, non-Hispanic families segregating for alcohol dependence, using one 10.3-cM microsatellite marker set and two SNP data sets (0.33-cM, 0.78-cM spacing). Two definitions of alcohol dependence (ALDX1 and ALDX2) were used. Our multipoint nonparametric linkage analysis found alcoholism was nominal linked to 12 genomic regions. The linkage peaks obtained by using the microsatellite marker set and the two SNP sets had a high degree of correspondence in general, but the microsatellite marker set was insufficient to detect some nominal linkage peaks. The presence of linkage disequilibrium between markers did not significantly affect the results. Across the entire genome, SNP datasets had a much higher average linkage information content (0.33 cM: 0.93, 0.78 cM: 0.91) than did microsatellite marker set (0.57). The linkage peaks obtained through two SNP datasets were very similar with some minor differences. We conclude that genome-wide linkage analysis by using approximately 5,000 SNP markers evenly distributed across the human genome is sufficient and might be more powerful than current 10-cM microsatellite marker assays.

## Background

In traditional linkage analysis for identifying genomic regions related to disease phenotypes, a whole-genome scan is usually performed using a set of 300–400 microsatellite markers evenly spaced across the genome. To maximize the chances of detecting linkage, the optimal amount of inheritance information is critical. This can be increased by genotyping more families and adding additional markers. With the rapid discovery of SNPs across the genome and the development of large-scale, high-throughput SNP genotyping approaches, high-density SNP assays throughout the genome may be a more rapid, powerful, and cost-effective tool than microsatellite marker assays in linkage analysis [1]. Recently, both simulation and applied studies have shown that high-density SNPs across the genome may offer several advantages over a low density microsatellite marker set, including increased power to detect linkage [2-4] and more precise mapping of the disease phenotype susceptibility loci [5]. The Collaborative Study on the Genetics of Alcoholism (COGA) data provided to participants in the Genetic Analysis Workshop 14 (GAW14) included one 10-cM microsatellite marker set and two high-density SNP genotype datasets, which offered a good opportunity to test the benefit of high-density SNPs relative to lower-density microsatellite markers in a whole-genome linkage scan.

## Methods

### Phenotype definition

The COGA dataset provided to participants in GAW14 was analyzed in this study. Only families with ethnicity self-reported as White, non-Hispanic were kept for analysis. Two diagnostic criteria for alcoholism were used in our analyses. For the first criterion, a diagnosis of alcoholism required positive diagnosis by the DSM-III-R criteria [6] and definite "alcoholism" by the Feighner criteria [7]. This is referred to as the COGA criterion for ALDX1. For the second criterion, a diagnosis of alcoholism only required positive diagnosis by the DSM-IV criterion [8], which is referred to as the COGA criterion for ALDX2. For each criterion, we classified individuals who are coded as "pure unaffected" under the COGA definition as unaffected. Individuals who showed some alcohol-related syndromes, but did not meet the criterion for affected and those who never drank alcohol were classified as "affection status unknown."

### Genetic maps and linkage disequilibrium

SNP genetic map positions were interpolated on the deCODE genetic map [9] through use of their physical positions (NCBI genome build 34.3); markers not placed were discarded. Since strong linkage disequilibrium (LD) might exist among some of the closely spaced SNPs and LD between SNPs might generate inflated linkage signals, we used Haploview (version 3.0) [10] to define LD blocks (default method) and selected only one tagging SNP with the highest heterozygosity among SNPs within each defined block.

### Linkage analysis

We performed multipoint nonparametric linkage analysis using an affected-only allele-sharing method, which was implemented in the ALLEGRO (version 1.2c) software [11]. We employed the *S*_pairs _scoring function [12], which performs well for all disease models, and the exponential allele-sharing model [13] to generate the relevant test statistics. Family scores were combined to obtain an overall score, using a weighting scheme that each family should be weighted proportionally to the standard deviation of the score function used, under the null hypothesis of no linkage, to the power 0.5, which is considered about midway between weighting each pair equally versus weighting each family equally [14].

## Results

**Figure 1:**
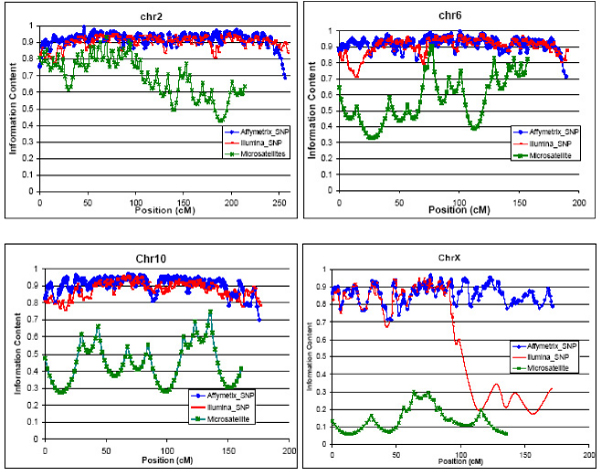
Linkage information content of high-density SNPs vs. microsatellites.

We used 115 White, non-Hispanic families in our analysis. The total number of individuals was 1,245, of which 1,009 were genotyped. Linkage information content for two SNP datasets was very similar except that the less-dense Illumina set had lower linkage information content on the X chromosome due to its poor coverage (Figure 1). Both SNP datasets had significantly higher linkage information content and better coverage than microsatellite marker data throughout the entire genome (Table 1).

For both definitions of alcohol dependence (ALDX1 and ALDX2), we found 12 genomic regions with nominally significant LOD scores (*p *< 0.05, Table 2). There was good concordance between the two SNP datasets in linkage peaks, except for the second peak on chromosome 6. We detected the linkage peaks discovered by the microsatellite marker assay with slightly higher LOD scores in both SNP datasets, with the exception of one peak on chromosome 21. We also detected two additional linkage peaks in both SNP datasets that were missed in microsatellite assay. This was likely due to low linkage information content (chromosome X) or poor coverage (chromosome 6).

Impact of the presence of LD was investigated by using the Affymetrix SNPs set, which had many LD blocks across the genome, and the results were not significantly changed when the analysis was restricted to SNPs in linkage equilibrium compared with the analysis without considering LD (Table 2).

## Discussion

This study supports the benefit of using of a high-density SNP marker set compared with a microsatellite marker assay in linkage analysis. Although there were only minor differences between the results from the two scans, the traditional microsatellite approach failed to detect some nominal linkage peaks due to lower linkage information content and poor coverage. The peaks on chromosome 6 (6q27) and X (Xp22) in the SNP assays were two examples of signals not detected in the microsatellite analyses. The good concordance between the two SNP marker sets (Affymetrix and Illumina) in both linkage information content and linkage findings suggests that >5,000 SNPs may be excessive for samples with structures similar to the COGA data, and a SNP scan with ~5,000 markers distributed evenly across the human genome is sufficiently dense and powerful in whole-genome linkage analysis. Also, with current technology SNP genotyping is more rapid, requires fewer samples, and is more accurate than microsatellite marker genotyping. High-density SNP marker sets also offer a better localization of linkage peaks, which may save work for fine mapping in regions showing linkage [4]. Since bi-allelic SNP markers are less informative than polymorphic microsatellite markers, the multipoint method is a better choice for SNP assays. However, estimation of genetic maps for SNPs is less precise than for microsatellite markers due to their lower levels of heterozygosity [15]. The computational burden increases dramatically as the number of markers increases. These disadvantages might limit the use of SNPs in whole-genome linkage scans.

Our analysis found nominal linkage for alcoholism to 12 genomic regions under both definitions for alcohol dependence (ALDX1 and ALDX2). The results for the two phenotype definitions are somewhat different. It is not clear which criterion is best for identifying genetic susceptibility loci for alcoholism. However, if one genomic region is associated with alcoholism, there should be similar statistical evidence under both criteria. Our finding on chromosome 2 overlaps with that of Reich et al. [16], who reported linkage of alcoholism to 2q13. Two important alcohol-related enzymes are located close to chromosomal regions where we found nominal linkage: the aldehyde dehydrogenase 2 family (ALDH2) is located on 12q24.2 and the cytochrome P450, family 2, subfamily E, polypeptide 1 (CYP2E1) is in 10q24.3–10q26.3 (Table 2). Our finding on chromosome X (Xp22), which showed evidence of linkage to mental retardation [17], sounds interesting for further investigation to explore gender differences for alcoholism.

## Conclusion

We conclude that a high-density SNP scan may offer a more rapid, cost-effective and powerful tool in genome-wide linkage analysis compared to traditional 10-cM microsatellite marker scans. However, further investigation is warranted to explore the effects of genetic map and computational issues on the utility of high density SNP assays in linkage analysis.

## Abbreviations

COGA: Collaborative Study on the Genetics of Alcoholism

GAW14: Genetic Analysis Workshop 14

LD: Linkage disequilibrium

SNP: Single-nucleotide polymorphism

## Authors' contributions

QM reconstructed the genetic map, carried out statistical analysis and drafted the manuscript. YY participated in genetic map reconstruction. YM and JF managed the data. LAF supported this study and helped to draft the manuscript. MAW conceived of the study, and participated in its design and helped to draft the manuscript. All authors read and approved the final manuscript.

## Figures and Tables

**Table 1:** Marker information.

**Dataset**	**Number**	**Average spacing (SD)**	**Information content (SD)**
Affymetrix SNPs	11,115	0.326 cM (0.615)	0.93 (0.037)
Illumina SNPs	4,720	0.775 cM (1.173)	0.91 (0.044)
Microsatellites	328	10.316 cM (7.656)	0.57 (0.18)

**Table 2:** Maximal LOD scores for loci with increased allele sharing at *p *< 0.05.

	**ALDX1 LOD (*p*)**				**ALDX2 LOD (*p*)**		
				
	**Affymetrix**		**Illumina**	**Microsatellites**	**Affymetrix**	**Illumina**	**Microsatellites**
						
	**LD**	**No LD**					
Chr 2 (2p25)	1.38 (0.005)	1.28 (0.007)	1.84 (0.002)	1.19 (0.009)	0.65 (0.04)	1.11 (0.01)	0.79 (0.03)
Chr 2 (2q13)	1.88 (0.002)	1.75 (0.002)	1.26 (0.008)	0.94 (0.02)	1.16 (0.01)	0.78 (0.03)	1.17 (0.01)
Chr 3 (3p21)	1.18 (0.01)	1.24 (0.008)	1.30 (0.007)	0.47 (0.07)	1.10 (0.01)	0.95 (0.02)	0.66 (0.04)
Chr 6 (6p24)	1.26 (0.008)	1.07 (0.01)	1.25 (0.008)	0.65 (0.04)	1.57 (0.003)	1.17 (0.01)	0.12 (0.2)
Chr 6 (6q27)	0.79 (0.03)	0.65 (0.04)	2.41 (0.0004)	-	0.95 (0.02)	2.08 (0.001)	-
Chr 7 (7p21)	1.69 (0.002)	1.64 (0.003)	1.15 (0.01)	1.16 (0.01)	1.84 (0.002)	1.40 (0.005)	1.01 (0.01)
Chr 9 (9q34)	0.90 (0.02)	0.81 (0.03)	1.17 (0.01)	0.28 (0.1)	1.53 (0.004)	1.72 (0.002)	0.08 (0.3)
Chr 10 (10q24)	1.02 (0.01)	0.62 (0.04)	1.21 (0.009)	0.91 (0.02)	1.83 (0.002)	2.43 (0.0004)	0.90 (0.02)
Chr 10 (10q26)	1.58 (0.003)	1.35 (0.007)	1.43 (0.005)	0.96 (0.02)	1.23 (0.008)	1.47 (0.004)	0.71 (0.03)
Chr 12 (12q24)	1.55 (0.004)	1.00 (0.02)	1.24 (0.008)	1.56 (0.004)	1.57 (0.003)	1.33 (0.006)	1.71 (0.002)
Chr 21 (21q22)	0.28 (0.1)	0.20 (0.2)	0.28 (0.1)	1.77 (0.002)	0.29 (0.1)	0.13 (0.2)	1.09 (0.01)
Chr X (xp22)	2.22 (0.0007)	-	2.17 (0.0007)	0.43 (0.08)	0.74 (0.03)	0.80 (0.03)	0.07 (0.3)
